# Impact of Region-of-Interest Size on the Diagnostic Performance of Shear Wave Elastography in Differentiating Thyroid Nodules

**DOI:** 10.3390/cancers15215214

**Published:** 2023-10-30

**Authors:** Kai-Lun Cheng, Pin-Hsien Lai, Chun-Lang Su, Jung Hwan Baek, Hsiang-Lin Lee

**Affiliations:** 1 Department of Medical Imaging, Chung Shan Medical University Hospital, Taichung 40201, Taiwan; chengkailun108@gmail.com (K.-L.C.); zardard@hotmail.com (P.-H.L.); 2 School of Medicine, Chung Shan Medical University, Taichung 40201, Taiwan; 3 Chung Jen Junior College of Nursing, Health Science and Management, Chiayi City 60077, Taiwan; s901055@gmail.com; 4 Department of Rehabilitation, Tung Wah Hospital, Nantou City 55713, Taiwan; 5 Department of Radiology and Research Institute of Radiology, Asan Medical Center, University of Ulsan College of Medicine, Seoul 05505, Republic of Korea; radbaek@naver.com; 6 Department of Surgery, Chung Shan Medical University Hospital, Taichung 40201, Taiwan

**Keywords:** thyroid nodule, ultrasound, shear wave elastography, region of interest, diagnostic performance, cancer diagnosis

## Abstract

**Simple Summary:**

In our pursuit for accurate thyroid nodule diagnosis, a common challenge, this study was conducted to investigate the role of shear wave elastography (SWE). Specifically, we examined the influence of the selection of different regions of interest (ROIs) on the diagnostic effectiveness of SWE. By employing three ROI sizes (1 mm, 2 mm, and Max), we found that although SWE is generally effective in distinguishing between malignant and benign nodules, the choice of ROI size can significantly influence the diagnostic accuracy of specific SWE elasticity metrics. Our study not only enhances the current understanding of SWE’s utility in thyroid nodule assessment but also offers valuable insights for future research, potentially paving the way for more reliable diagnostic techniques.

**Abstract:**

This study investigated the impact of different region-of-interest (ROI) sizes (Max, 1 mm, and 2 mm) on shear wave elastography (SWE) in differentiating between malignant and benign thyroid nodules. The study cohort comprised 129 thyroid nodules (50 malignant, 79 benign) and 78 normal subjects. Diagnostic efficacy was assessed through pairwise comparisons of area under the curve (AUC) values in receiver operating characteristic analysis by using DeLong’s test. Our results indicated significant differences in all SWE elasticity metrics between the groups, with malignant nodules exhibiting higher values than benign nodules (*p* < 0.05). Smaller ROIs (1 and 2 mm) were found to outperform the max ROI in terms of diagnostic accuracy, particularly for the *E_max_* and *E_min_* elasticity metrics. *E_max(_*_1mm*)*_ had the highest diagnostic accuracy, with an AUC of 0.883, sensitivity of 74.0%, and specificity of 86.1%. This study underscores the significant influence of ROI size selection on the diagnostic performance of SWE, offering valuable insights for future research and clinical applications in thyroid nodule assessment.

## 1. Introduction

Shear wave elastography (SWE) is an advanced elastography technique that is used to quantitatively evaluate tissue stiffness, a factor that is crucial for distinguishing between benign and malignant lesions. In this method, shear waves are generated through the effective vibration of tissue particles, which is initiated by an acoustic radiation pulse emitted by a transducer [1]. These shear waves are subsequently employed to calculate the Young’s modulus, measured in kilopascals (kPa). SWE minimizes dependence on the skills of the individual operator, leading to high reproducibility and standardized quantitative assessment [2]. Consequently, SWE has emerged as a preferred approach, particularly valued for its quantitative capability in differentiating between various types of lesions, including those occurring in the thyroid [3,4,5,6,7,8,9], breast [10,11,12], and lymph nodes [13,14]. A meta-analysis conducted by Filho et al. [15] examined the use of SWE devices from various manufacturers to distinguish between benign and malignant thyroid nodules. Their findings revealed sensitivities ranging from 63% to 77%, specificities between 76% and 81%, and area under the receiver operating characteristic (ROC) curve (AUC) values ranging from 0.84 to 0.88. They concluded that SWE is an essential tool that can complement ultrasound (US) in clinical practice for the diagnostic differentiation between benign and malignant thyroid nodules.

The establishment of universally applicable diagnostic criteria remains challenging, primarily because SWE results can be influenced by various factors. The selection of the size of regions of interest (ROIs) has emerged as a significant contributor to SWE’s performance [16]. This contribution has been explored in the context of breast [10,11,12] and lymph node [13,14] assessments. In the field of thyroid disease research, various parameters and cutoff values have been employed, leading to methodological inconsistencies. Currently, no standardized method for determining ROIs is available. Some researchers have selected ROIs with diameters of 1–3 mm [6,7,9,17,18], whereas others have favored large ROIs that encompass the entire lesion [8,19]. The lack of a standard ROI determination method underscores the complexity of achieving consistent SWE implementation across different studies and clinical settings.

To date, studies comparing the two types of ROIs—small ROIs targeting the lesion and large ROIs encompassing the entire lesion—have been limited. To address this gap, the present study quantitatively examined the impact of three ROI sizes on SWE elasticity metrics and diagnostic performance in differentiating between malignant and benign thyroid nodules.

## 2. Materials and Methods

The authors were accountable for all aspects of this research, ensuring that any issues regarding the accuracy or integrity of any part of the study were diligently examined and resolved. The study protocol adhered to the principles outlined in the Declaration of Helsinki. The study was approved by the Institutional Review Board (IRB) of Chung Shan Medical University Hospital (IRB Approval No.: CS2-23095), and individual consent was waived due to the study’s retrospective nature.

### 2.1. Study Population

We conducted a retrospective analysis of consecutive patients who presented with solid thyroid nodules larger than 5 mm in diameter at a single center between November 2022 and June 2023. The following patients were included in the study: (1) patients aged ≥ 18 years, (2) individuals who had undergone both two-dimensional and SWE US examinations prior to fine-needle aspiration (FNA), and (3) patients who had received FNA and whose cytological results corresponded to Bethesda category II (benign), V (suspicious for malignancy), or VI (malignant). FNA was recommended when there were suspicious clinical and US features, including the presence of microcalcifications, a taller-than-wide nodule shape, or a spiculated/microlobulated margin [20]. Additionally, FNA was performed when a patient requested it for reassurance, even in the absence of suspicious findings. The exclusion criteria were as follows: (1) a history of thyroidectomy or lobectomy, (2) the presence of multinodular disease without distinct isolated nodules, (3) inconclusive FNA cytological diagnosis (Bethesda category I, III, or IV), and (4) suboptimal quality of images from the SWE US examination. Malignant nodules were defined on the basis of cytological results falling into Bethesda category V or VI. In cases where patients initially presented for evaluation due to suspected thyroid nodules, regions exhibiting a typical appearance of normal thyroid parenchyma were identified during US examination and used as representative samples for the normal subjects. All enrollees exhibited euthyroid status. The demographic data of the enrollees are presented in Table 1.

### 2.2. SWE Evaluation and Measurement

For this study, SWE images and data from patients seen during the aforementioned timeframe were collected and subsequently reanalyzed for our research objectives. The thyroid ultrasound (US) and 2D-SWE scans associated with these images were originally conducted using a Samsung RS80 EVO machine (Samsung Medison, Seoul, Republic of Korea) equipped with a 2–14 MHz linear-array probe (LA2-14A). All thyroid US imaging was performed by a board-certified neuroradiologist (KL Cheng) who possessed over 15 years of experience in the field. To achieve uniformity in the study, the investigator standardized the machine settings by using the same thyroid US scanning preset and adhering to established US scanning protocols.

**Figure 1:**
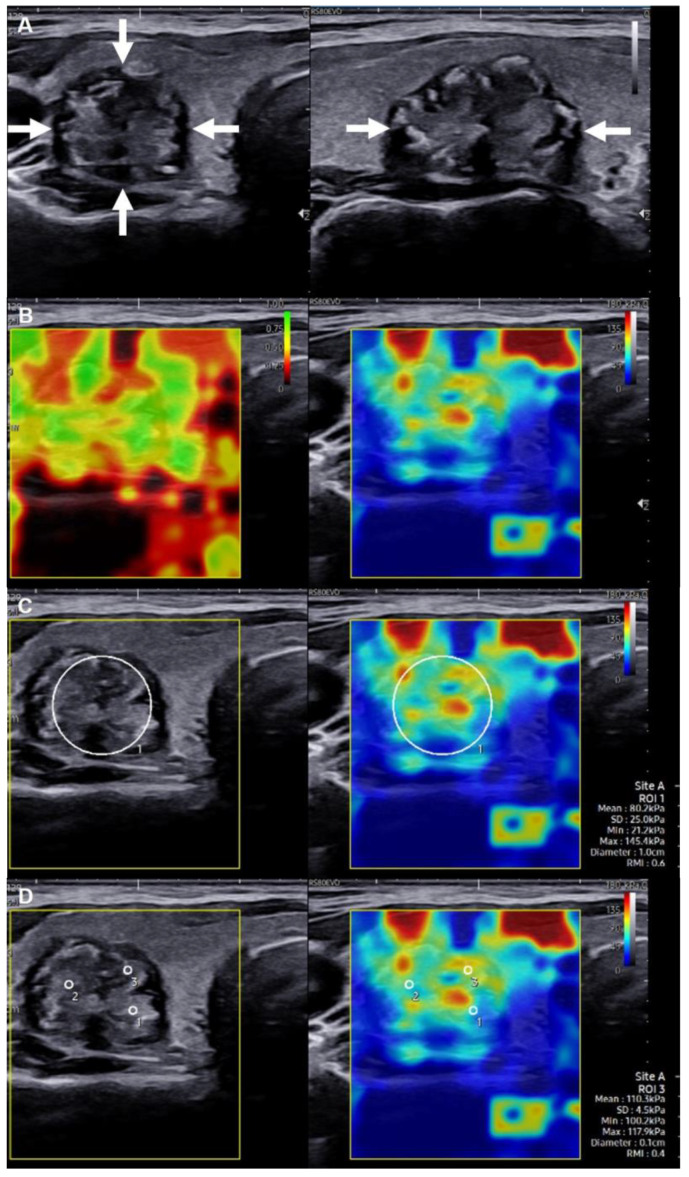

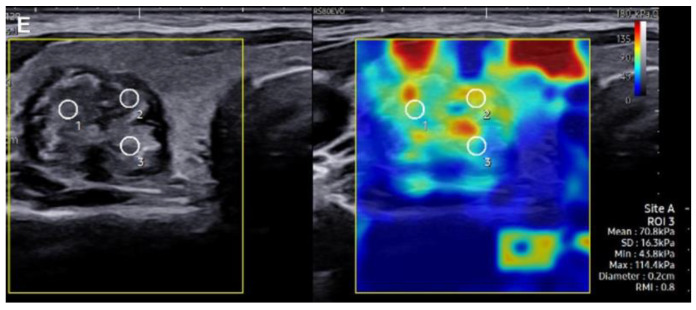
Selection of regions of interest (ROIs) of three sizes in a 46-year-old female patient with a Bethesda category VI nodule (papillary thyroid cancer): (**A**) Grayscale US image revealing a hypoechoic solid nodule in the right thyroid lobe and with irregular margins (indicated by arrows); the nodule measures 11.4 × 13.5 × 18.5 mm. (**B**) Reliability measurement index (RMI) map, which consists of a semitransparent color map overlaid on a grayscale image. The color scale ranges from dark red, indicating the lowest value, to green, indicating the highest value within the 0–1 range. Right: Shear wave elastography (SWE) map, displaying a semitransparent color map of tissue stiffness overlaid on a grayscale image. The color scale ranges from dark blue, indicating the lowest stiffness, to red, indicating the highest stiffness within the 0–180-kPa range. (**C**) Max ROI: A circular ROI is adjusted to conform to the lesion’s contours, covering the maximum area of the nodule. Three different circular ROIs with diameters of (**D**) 1 and (**E**) 2 mm selected on the basis of an RMI of ≥0.4.

For US imaging, the individual undergoing the imaging was positioned in a supine posture, with their neck gently extended over a pillow. Following the initial US examination, the transducer was switched to SWE mode. A liberal amount of coupling gel was applied to the individual’s neck, and the probe was placed as lightly as possible on the lesion to minimize compression artifacts. Care was taken to ensure that the probe remained motionless during image acquisition. The individual was briefly instructed to hold their breath to minimize potential interference. Once a stable image had been acquired, the SWE sampling box, with maximum dimensions of 23 mm × 25 mm, was adjusted to encompass as much of the entire thyroid nodule as possible. The SWE sampling box enabled the placement of circular ROIs within the elastograms, with a summary of the stiffness data automatically displayed. Three SWE ROIs were defined as follows (Figure 1): (1) max, where a circular ROI was adjusted to match the lesion contours, capturing the largest possible area of the nodule; (2) 2 mm, represented by a circular ROI with a diameter of 2 mm; and (3) 1 mm, represented by a circular ROI with a diameter of 1 mm. In Samsung’s SWE technology, a specialized index, known as the reliability measurement index (RMI), is employed to enhance the reliability of shear-wave elasticity assessments [21,22,23]. The RMI is calculated using a combination of the residual of the wave equation and the amplitude of the shear wave [24]. The RMI ranges from 0.0 to 1.0, with an RMI of 1.0 considered the gold standard for measurement consistency. The technology also provides a dual-map display (Figure 1B) that comprises both stiffness and RMI maps, thereby facilitating an intuitive and reliable assessment of tissue elasticity. Nonetheless, no manufacturer-specified RMI guidelines specifically tailored to thyroid studies are available. Therefore, for the 2 mm and 1 mm ROIs, three distinct areas within the lesion were selected on the basis of RMI ≥ 0.4, in accordance with the literature [25,26]. The average values derived from these three ROIs were then used to determine the final value [4,27]. In the case of the max ROI, a single region within the lesion was selected for measurement.

**Figure 2:**
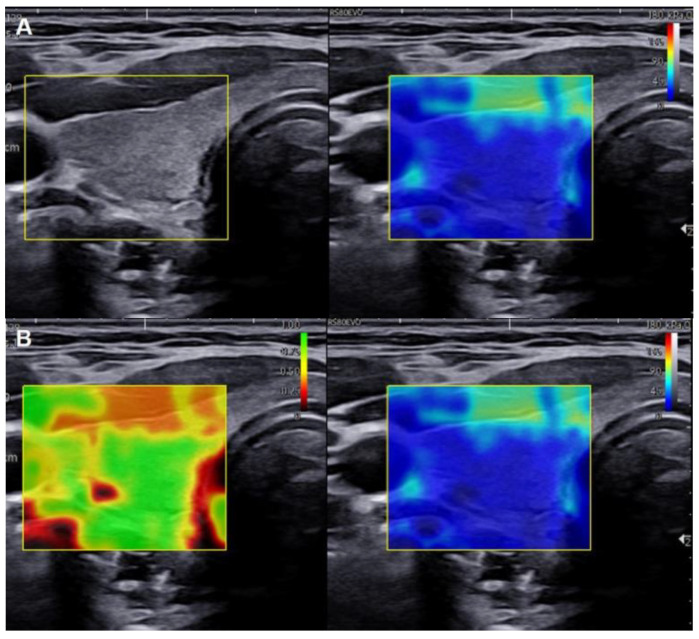

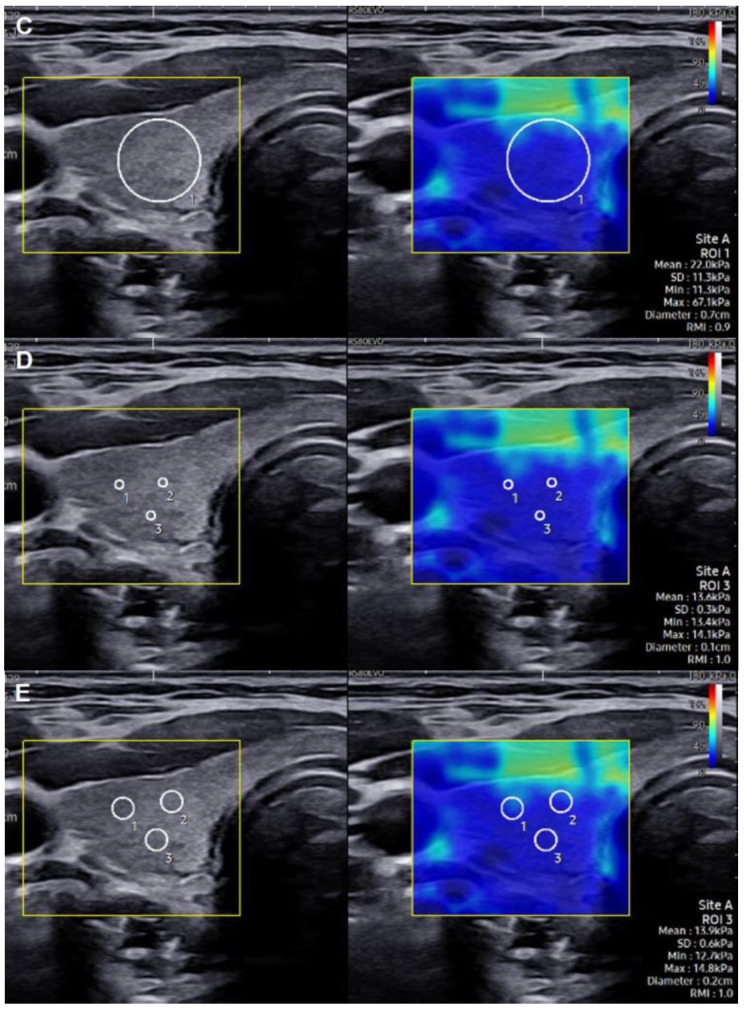
Selection of regions of interest (ROIs) of three sizes in a 26-year-old female patient with right normal thyroid parenchyma: (**A**) Grayscale US image showing the typical appearance of normal thyroid parenchyma in the right thyroid lobe. (**B**) (**Left**): Reliability measurement index (RMI) map, which consists of a semitransparent color map overlaid on a grayscale image. The color scale ranges from dark red, indicating the lowest value, to green, indicating the highest value within the 0–1 range. (**Right**): Shear wave elastography (SWE) map, displaying a semitransparent color map of tissue stiffness overlaid on a grayscale image. The color scale ranges from dark blue, indicating the lowest stiffness, to red, indicating the highest stiffness within the 0–180 kPa range. (**C**) Max ROI: A circular ROI adjusted to encompass as much of the normal thyroid parenchyma as possible. Three different circular ROIs with diameters of 1 mm (**D**) and 2 mm (**E**), each capturing representative sections of the normal thyroid parenchyma based on an RMI of ≥0.4.

This study recorded SWE elasticity metrics, which included the maximum (*E_max_*), mean (*E_mean_*), minimum (*E_min_*), and standard deviation (*E_SD_*) elasticity values for all ROIs, in kilopascals (kPa). The same methodology was applied to normal subjects to determine the SWE elasticity metrics for the normal thyroid parenchyma (Figure 2). To evaluate intraobserver agreement, the same investigator repeated measurements on a randomly selected subset of 20 cases. This subset included both malignant (Bethesda category V or VI) and benign (Bethesda category II) nodules. The repeated measurements were conducted with a 1-month interval between measurements [28]. 

### 2.3. Statistical Analysis

Categorical variables are presented as raw counts, whereas continuous variables are presented as the mean ± standard deviation (SD), median, and range. The normality of the distribution of continuous variables was assessed using the Kolmogorov–Smirnov test.

On the basis of the results of normality testing, either a one-way analysis of variance (ANOVA) or the Kruskal–Wallis test was employed, along with an appropriate post hoc test (Student–Newman–Keuls or the Conover method), to compare age and SWE elasticity metrics across the three ROIs within the target nodules in the malignant, benign, and normal subjects groups. The chi-squared test was employed to compare the sex distribution between the target nodules and the normal subjects. The Mann–Whitney U test was used to assess differences in maximum diameter and volume between malignant and benign nodules. 

To assess the diagnostic performance of significant predictive values derived from SWE elasticity metrics, an ROC analysis was conducted. The optimal threshold for the significant predictive value of malignant nodules was confirmed by weighting sensitivity and specificity equally on the ROC curve. To verify the differences in diagnostic performance between SWE elasticity metrics obtained from the three ROIs, pairwise comparisons between the AUCs of these ROC curves were performed using DeLong’s test. 

Intraobserver agreement was examined using intraclass correlation coefficients (ICCs). These coefficients are interpreted as follows: <0.5, 0.5–0.75, 0.75–0.90, and >0.90 indicate poor, moderate, good, and excellent agreement, respectively [29]. 

All statistical tests performed in this study were two-sided, and a *p*-value of <0.05 was considered statistically significant. The statistical analyses were conducted using MedCalc^®^ Statistical Software version 20.014 (MedCalc Software Ltd., Ostend, Belgium; https://www.medcalc.org (accessed on 19 July 2023).)

## 3. Results

### 3.1. Patients

In this study, we analyzed 129 thyroid nodules from 129 patients, which included 79 benign and 50 malignant nodules, along with 78 normal subjects. Of the 50 malignant nodules, 36 were diagnosed as papillary thyroid cancer (Bethesda category VI), whereas 14 were classified as suspicious for papillary thyroid cancer (Bethesda category V). Notably, no significant differences in terms of sex or age were discovered between the malignant nodules, benign nodules, and normal subjects. The benign nodules had a significantly larger maximum diameter (mean ± SD: 25.6 ± 12.5 mm) and volume (mean ± SD: 6.7 ± 8.1 mL) than the malignant nodules (mean diameter = 12.9 ± 9.1 mm and volume = 1.9 ± 6.1 mL; both *p* < 0.05). 

### 3.2. Comparison of SWE Elasticity Metrics in the Malignant Nodules, Benign Nodules, and Normal Subjects Groups

Table 2 presents the SWE elasticity metric parameters (*E_max_*, *E_min_*, *E_mean_*, and *E_SD_*) for the malignant nodules, benign nodules, and normal subjects. Notably, significant intergroup differences were found in all SWE elasticity metrics (all *p* < 0.05). The results of the post hoc analysis revealed that all SWE elasticity metrics for malignant nodules were significantly higher than those for benign nodules (all *p* < 0.05). The intraobserver agreement for *E_SD_*_(2mm)_ was found to be moderate (ICC = 0.65), whereas that for the other indices was good–excellent (ICC values = 0.76–1.00).

### 3.3. Comparison of SWE Elasticity Metrics in Three ROIs in the Malignant Nodules, Benign Nodules, and Normal Subjects Groups

Table 2 provides a comprehensive overview of the results of the ROI comparison. Regarding the *E_mean_* values, no significant differences were observed between the three ROIs in any group. By contrast, for all groups, the *E_max_*, *E_min_*, and *E_SD_* values obtained for the three ROIs were significantly different. Post hoc analysis revealed that the *E_max_*_(1mm)_ and *E_max_*_(2mm)_ values were significantly lower than the *E_max_*_(Max)_ value in all groups. However, the differences between *E_max_*_(1mm)_ and *E_max_*_(2mm)_ were nonsignificant for the malignant and benign nodules groups. Regarding *E_min_*, the *E_min_*_(1mm)_ and *E_min_*_(2mm)_ values were significantly higher than the *E_min_*_(Max)_ value for all groups; however, the differences between *E_min_*_(1mm)_ and *E_min_*_(2mm)_ were nonsignificant. Regarding *E_SD_*, the pairwise comparisons indicated significant variation among the three ROIs for all groups, with *E_SD_*_(Max)_ consistently being highest.

### 3.4. Assessment of the Diagnostic Performance of SWE Elasticity Metrics in Distinguishing between Malignant and Benign Nodules

The diagnostic efficacy of the SWE elasticity metrics was evaluated through ROC analysis, and the results are summarized in Table 3. All SWE elasticity metrics achieved statistical significance (*p* < 0.05), underscoring their strong potential in distinguishing between malignant and benign nodules. Notably, *E_max_*_(1mm)_ yielded the highest AUC of 0.883, sensitivity of 74.0%, specificity of 86.1%, and a designated cutoff value of 61.4 kPa.

**Figure 3:**
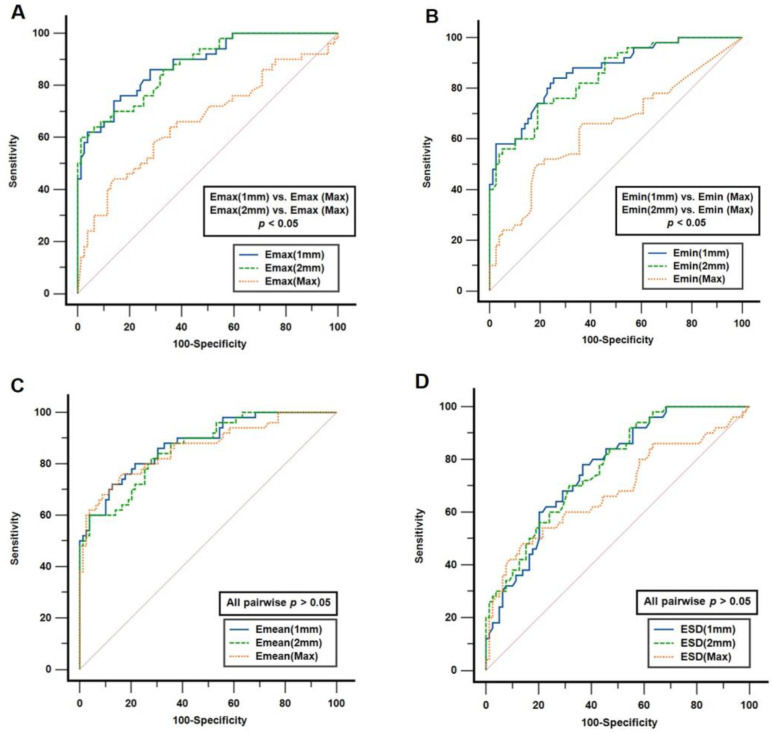
Comparison of diagnostic performance in predicting malignant nodules among (**A**) *E_max_*, (**B**) *E_min_*, (**C**) *E_mean_* and (**D**) *E_SD_* derived from the three ROIs.

Further analysis was conducted to compare the diagnostic performance of the SWE elasticity metrics across the three distinct ROIs, as depicted in Figure 3. The *E_max_* and *E_min_* values derived from the 1 mm and 2 mm ROIs demonstrated superior diagnostic capability compared with those derived from the max ROI (*E_max_* AUC: 0.883 (1 mm) vs. 0.664 (max), *p* < 0.05; 0.877 (2 mm) vs. 0.664 (max), *p* < 0.05. *E_min_* AUC: 0.867 (1 mm) vs. 0.646 (max), *p* < 0.05; 0.846 (2 mm) vs. 0.646 (max), *p* < 0.05]. However, no significant differences were noted in the diagnostic outcomes of *E_mean_* and *E_SD_* across the three ROIs (all pairwise comparisons yielded *p* > 0.05).

## 4. Discussion

In the present study, the three groups (malignant nodule, benign nodule, and normal subjects) had significantly different SWE elasticity metrics derived from the three ROIs. These metrics were significantly higher in the malignant nodules group than in the benign nodules group, which is consistent with the general findings reported in the literature. For all three groups, the values of *E_max_*, *E_min_*, and *E_SD_* (but not *E_mean_*) were significantly different for the three ROIs (Max, 1 mm, and 2 mm). Nevertheless, all SWE elasticity metrics demonstrated a strong ability to distinguish between malignant and benign nodules. We compared the diagnostic performance of the SWE elasticity metrics across three ROIs. For the metrics *E_max_* and *E_min_*, the 1 mm and 2 mm ROIs showed better results than the max ROI. However, *E_mean_* and *E_SD_* showed no significant differences across the ROIs. Notably, *E_max_*_(1mm)_ had the highest AUC at 0.883 (95% confidence interval: 0.814–0.933). Its optimal cutoff value was 61.4 kPa, with a sensitivity of 74.0% and specificity of 86.1%.

The impact of ROIs in SWE for thyroid nodule evaluation has not been systematically explored in previous research. Various ROI sizes and placements have been employed in the evaluation of breast masses [10,11,12] and lymph nodes [13,14]. In thyroid-related studies, the selection of ROIs has varied widely. Some researchers have selected large ROIs that covered an entire lesion [8,19], whereas others have favored small, targeted ROIs with diameters between 1 and 3 mm [6,7,9,17,18]. Additionally, some studies have adopted a mixed approach, which involves using both large and small ROIs [4,30,31]. Smaller ROIs have been demonstrated to be more effective in differentiating benign from malignant lesions in thyroid [4], breast [10], and lymph node [13] examinations. A meta-analysis conducted by Suh et al. suggested that the choice of ROI could contribute to the variation in diagnostic performance observed in SWE applications for cervical lymph nodes [16]. Therefore, an investigation into the influence of ROI size on elasticity metrics is crucial for enhancing the precision of thyroid nodule diagnosis through SWE. In this study, we conducted a comparative analysis of three distinct ROI sizes: two small circular ROIs with different diameters, and one maximal ROI that delineated the entire nodule as comprehensively as possible. 

Our study’s findings highlight the impact of ROI selection on both elasticity metrics and diagnostic performance in the context of SWE-based thyroid nodule evaluation. For each elasticity metric, significant differences were observed between malignant nodules, benign nodules, and normal subjects, regardless of the ROI selected. Subsequent post hoc analysis revealed that malignant nodules consistently exhibited significantly higher values than benign nodules did, reaffirming the ability of elasticity metrics to differentiate between benign and malignant nodules. This outcome is consistent with previous findings [7,8,9]. However, *E_max_*, *E_min_*, and *E_SD_* were significantly different between the various ROIs not only in malignant or benign nodules, but also in normal subjects. In terms of diagnostic performance in differentiating between benign and malignant nodules, smaller ROIs were discovered to have superior diagnostic performance when considering *E_max_* and *E_min_*. However, regarding *E_mean_* and *E_SD_*, no significant differences in diagnostic efficacy were found between the different ROIs. The results of the present study indicate that the choice of ROI can have a considerable impact on the measurement of elasticity metrics and, consequently, on the diagnostic performance of SWE. Therefore, future research should establish clear and standardized ROI selection criteria to minimize potential biases. 

Of the SWE elasticity metrics, *E_max_* has gained recognition as a valuable predictor of malignant nodules [6,30,31,32]. However, the optimal threshold for using *E_max_* values in SWE to differentiate between malignant and benign nodules has been inconsistent across studies, leading to varying diagnostic outcomes. For instance, Veyrieres et al. identified a cutoff value of 66 kPa for *E_max_* as optimal for distinguishing malignant nodules; the associated sensitivity was 80%, the specificity was 90.5%, and the AUC was 0.852 [5]. Katarzyna et al. identified a similar cutoff value of 67.0 kPa, although this was found to yield a lower sensitivity of 42.0% and specificity of 88.2% [30]. By contrast, Park et al. proposed a higher cutoff value—94 kPa, which yielded a sensitivity of 46.4% and specificity of 85.6% [6]. On the basis of an ROC curve analysis, Tan et al. suggested that *E_max_* ≥ 28.2 kPa was the optimal threshold [32]. In the context of the present study, *E_max_*_(1mm)_ exhibited the highest AUC of 0.883, with the optimal cutoff being 61.4 kPa, sensitivity being 74.0%, and specificity being 86.1%. The discrepancies in these *E_max_* cutoff values across studies could be due to various factors, including differences in sample sizes, nodule dimensions, and benign-to-malignant ratios within the study groups.

In our examination of the *E_max_* data, we discovered that the values for *E_max_*_(Max)_ were significantly higher than those for *E_max_*_(1mm)_ and *E_max_*_(2mm)_. Studies have often placed the ROI in the stiffest region of a nodule [4,6,13,14,30], frequently resulting in smaller ROIs being positioned in the stiffest area. This practice has led to *E_max_* values that are similar to or even higher than those obtained with larger ROIs [13,14]. However, our approach to ROI selection was different; instead of targeting the stiffest part of a nodule, we based our ROI selection on RMI values. The RMI serves as a quality assurance metric, capturing both a signal’s strength and the level of noise or potential motion artifacts in each individual reading. A higher RMI indicates either peak signal strength or minimal noise, making the RMI a reliable indicator of measurement accuracy. Preliminary research has demonstrated that high RMI values are strongly correlated with reproducible measurements [24]. In liver studies, RMI values ranging from 0.4 to 0.8 have been employed [33,34,35]. However, the thyroid presents different conditions compared with the liver, and specific recommended RMI values for thyroid research were not provided by the manufacturer. Therefore, we adhered to the standards established in studies on salivary glands [26] and cervical lymph nodes [25], and we selected an RMI of ≥0.4 as the criterion. Our results suggest that selecting the appropriate RMI region does not necessarily result in targeting of the stiffest part of the nodule. This distinction could account for the differences between our data and those from other studies. Nevertheless, the SWE values obtained through our ROI selection method effectively differentiated between benign and malignant nodules, exhibiting commendable diagnostic performance. Additionally, our approach resulted in moderate-to-good intraobserver agreement. Therefore, our findings indicate that an RMI value of ≥0.4 may serve as a standard for selecting the ROI in thyroid nodule SWE assessments in the future.

Our study has some limitations that must be acknowledged. As a retrospective analysis conducted on patients from a specific timeframe, there are inherent limitations such as potential selection bias and the inability to control for certain variables. First, this study did not compare the diagnostic performance of different thyroid imaging report and data systems and traditional US features for thyroid nodules. Although these features are fundamental diagnostic tools and play a crucial role in estimating the risk of malignancy, they have been extensively studied. Therefore, our research was focused on the impact of different ROIs on SWE and discovered that the three ROIs affected the diagnostic performance. Second, in addition to including thyroid nodules classified as Bethesda category VI (malignant), we also included those in Bethesda category V (suspicious for malignancy). Although these categories represent distinct malignancy risk levels, the 2015 American Thyroid Association management guidelines recommend surgical management for both categories [36]. Moreover, studies have listed Bethesda category V (suspicion of malignancy) as indicative of thyroid malignancy [37]. Furthermore, not all nodules, especially those diagnosed as benign through FNA, were subjected to final histological confirmation—a factor that may have introduced bias into our findings. Third, extremely large lesions may have extended beyond the maximum elastographic overlay or field of view. Despite our efforts to select the most representative cross-section for SWE analysis, this limitation may have affected our results. Fourth, we did not explore other factors that might affect SWE, such as nodule size and whether the SWE measurements originate from transverse or longitudinal planes. Fifth, we did not assess interobserver agreement. Finally, the conclusions drawn regarding the utility of ROI selection are specific to the Samsung RS80 US device. Because SWE results may vary between different US devices, further research involving various manufacturers’ US devices would be necessary to corroborate our findings. 

## 5. Conclusions

This study underscored the potential of SWE in quantifying the elasticity of thyroid lesions and effectively differentiating between malignant and benign thyroid nodules. Through the use of circular ROIs of different sizes (max, 1 mm, and 2 mm), significant differences were observed in all SWE elasticity metrics between the groups, with malignant nodules exhibiting higher values than benign ones. Importantly, the selection of ROI size was found to have a profound impact on the diagnostic performance of SWE, with *E_max(_*_1mm*)*_ showing the highest diagnostic accuracy, as evidenced by an AUC of 0.883. These results emphasize the importance of careful ROI selection in leveraging the full diagnostic potential of SWE for thyroid nodule assessment and provide valuable insights for future research and clinical applications.

## Figures and Tables

**Table 1:** Demographic information of the study participants.

	Malignant (n = 50)	Benign (n = 79)	Normal Subjects (n = 78)	*p*
Sex (F:M)	38:12	63:16	57:21	0.62 ^C^
Age (y/o)	46.6 ± 10.5	48.3 ± 11.4	44.5 ± 11.9	0.19 ^A^
	44.0	47.0	44.5	
	(29–67)	(18–82)	(18–81)	
Max. dia. (mm)	12.9 ± 9.1	25.6 ± 12.5	-	<0.05 ^M^
	9.4	24.7		
	(5.8–49.6)	(6.5–50.2)		
Volume (mL)	1.9 ± 6.1	6.7 ± 8.1	-	<0.05 ^M^
	0.30	3.6		
	(0.06–41.7)	(0.06–35.1)		

Max. dia.: maximum diameter; ^C^: chi-squared test; ^A^: one-way ANOVA; ^M^: Mann–Whitney U test. Except for sex, all other data are presented as the mean ± standard deviation (SD), median, and range.

**Table 2:** SWE values from three ROIs in the malignant nodules, benign nodules, and normal subjects groups.

SWE Indices	Malignant (M) (n = 50)	ICC (95% CI)	Benign (B) (n = 79)	ICC (95% CI)	Normal Subjects (N) (n = 78)	*p*
*E_max_*_(Max)_	112.90 ± 43.69 105.20 (17.9–179.3)	1.00 (0.99–1.00)	89.26 ± 31.41 86.80 (28.20–179.3)	0.98 (0.95–0.99)	65.20 ± 31.18 61.40 (22.60–173.6)	<0.001 ^A^ (all pairwise *p* < 0.05) *
*E_max_*_(1mm_)	88.49 ± 37.50 87.80 (26.37–167.77)	0.92 (0.81–0.97)	37.72 ± 20.38 33.67 (5.40–91.73)	0.96 (0.90–0.98)	31.83 ± 13.98 29.03 (13.17–73.17)	<0.001 ^K^ (M vs. B; M vs. N) *
*E_max_*_(2mm)_	95.42 ± 36.72 94.17 (34.70–167.77)	0.96 (0.90–0.98)	45.15 ± 21.50 41.43 (11.07–94.83)	0.95 (0.87–0.98)	38.04 ± 16.85 33.88 (14.80–83.30)	<0.001 ^K^ (all pairwise *p* < 0.05) *
*p*	0.007 ^A^ (Max vs. 1 mm; Max vs. 2 mm) *		<0.001 ^A^ (Max vs. 1 mm; Max vs. 2 mm) *		<0.001 ^K^ (all pairwise *p* < 0.05) *	
*E_min_*_(Max)_	20.40 ± 19.03 18.70 (0.70–81.9)	0.97 (0.92–0.99)	11.11 ± 11.18 7.10 (0.70–48)	0.97 (0.92–0.99)	12.77 ± 5.39 12.20 (0.70–26.8)	0.002 ^K^ (B vs. M; B vs. N) *
*E_min_*_(1mm)_	69.85 ± 34.99 68.68 (15.50–146.33)	0.88 (0.73–0.95)	27.92 ± 15.62 24.53 (3.97–72.47)	0.97 (0.91–0.99)	24.34 ± 10.21 22.30 (9.67–56.93)	<0.001 ^K^ (M vs. B; M vs. N) *
*E_min_*_(2mm)_	57.01 ± 29.0 57.65 (13.87–124.73)	0.90 (0.77–0.96)	24.80 ± 14.13 20.93 (5.63–63.30)	0.95 (0.88–0.98)	21.88 ± 8.55 20.50 (7.53–45.87)	<0.001 ^K^ (M vs. B; M vs. N) *
*p*	<0.001 ^K^ (Max vs. 1 mm; Max vs. 2 mm) *		<0.001 ^K^ (Max vs. 1 mm; Max vs. 2 mm) *		<0.001 ^K^ (Max vs. 1 mm; Max vs. 2 mm) *	
*E_mean_*_(Max)_	67.19 ± 24.38 71.70 (22.90–118)	0.97 (0.93–0.99)	34.25 ± 15.04 32.40 (10.90–77.1)	0.99 (0.97–1.00)	28.48 ± 11.40 27.15 (11.80–59.3)	<0.001 ^K^ (all pairwise *p* < 0.05) *
*E_mean_*_(1mm)_	79.62 ± 36.66 77.68 (20.17–162.07)	0.89 (0.74–0.95)	32.67 ± 17.90 28.30 (4.57–77.90)	0.96 (0.89–0.98)	27.92 ± 11.89 25.15 (10.90–64.40)	<0.001 ^K^ (M vs. B; M vs. N) *
*E_mean_*_(2mm)_	76.75 ± 33.74 74.90 (23.47–143.43)	0.95 (0.88–0.98)	33.65 ± 17.63 30.70 (8.33–80.83)	0.95 (0.89–0.9891)	28.95 ± 12.01 26.00 (10.97–59.13)	<0.001 ^K^ (M vs. B; M vs. N) *
*p*	0.240 ^K^		0.566^K^		0.838^K^	
*E_SD_*_(Max)_	23.62 ± 14.61 19.80 (4.40–60.4)	0.99 (0.97–1.00)	14.75 ± 7.58 13.50 (3.60–51.5)	1.00 (0.99–1.00)	10.31 ± 6.62 9.30 (2.20–27.9)	<0.001 ^K^ (all pairwise *p* < 0.05) *
*E_SD_*_(1mm)_	4.79 ± 2.72 4.33 (1.33–13.37)	0.86 (0.69–0.94)	2.60 ± 1.95 2.13 (0.23–8.43)	0.89 (0.75–0.96)	1.90 ± 1.29 1.47 (0.40–5.27)	<0.001 ^K^ (all pairwise *p* < 0.05) *
*E_SD_*_(2mm)_	9.52 ± 5.72 7.87 (3.07–29.33)	0.65 (0.30–0.85)	5.02 ± 2.92 4.87 (0.63–14.03)	0.84 (0.63–0.93)	4.07 ± 2.70 3.18 (0.67–12.37)	<0.001 ^K^ (all pairwise *p* < 0.05) *
*p*	<0.001 ^K^ (all pairwise *p* < 0.05) *		<0.001 ^K^ (all pairwise *p* < 0.05) *		<0.001 ^K^ (all pairwise *p* < 0.05) *	

SWE: shear-wave elastography; ICC: intraclass correlation coefficient; ^A^: one-way ANOVA; ^K^: Kruskal–Wallis test. All data are presented as the mean ± standard deviation (SD), median, and range (in kPa). *: Significance after post hoc testing (Student–Newman–Keuls or the Conover method).

**Table 3:** Diagnostic performance of SWE elasticity metrics obtained from three ROIs in differentiating between malignant and benign nodules.

SWE Indices	AUC (95% CI)	Optimal Cutoff (kPa)	Sensitivity (%) (95% CI)	Specificity (%) (95% CI)
*E_max_*_(Max)_	0.664 (0.575–0.745)	>117.9	44.0 (30.0–58.7)	86.1 (76.5–92.8)
*E_max_*_(1mm_)	0.883 (0.814–0.933)	>61.4	74.0 (59.7–85.4)	86.1 (76.5–92.8)
*E_max_*_(2mm)_	0.877 (0.807–0.928)	>88.0	60.0 (45.2–73.6)	98.7 (93.1–100.0)
*E_min_*_(Max)_	0.646 (0.557–0.728)	>18.4	50.0 (35.5–64.5)	81.0 (70.6–89.0)
*E_min_*_(1mm)_	0.867 (0.797–0.921)	>37.0	84.0 (70.9–92.8)	74.7 (63.6–83.8)
*E_min_*_(2mm)_	0.846 (0.772–0.903)	>37.4	74.0 (59.7–85.4)	81.0 (70.6–89.0)
*E_mean_*_(Max)_	0.863 (0.791–0.917)	>50.6	76.0 (61.8–86.9)	83.5 (73.5–90.9)
*E_mean_*_(1mm)_	0.879 (0.810–0.930)	>54.8	72.0 (57.5–83.8)	87.3 (78.0–93.8)
*E_mean_*_(2mm)_	0.863 (0.791–0.917)	>64.2	60.0 (45.2–73.6)	96.2 (89.3–99.2)
*E_SD_*_(Max)_	0.682 (0.594–0.761)	>20.9	48.0 (33.7–62.6)	86.1 (76.5–92.8)
*E_SD_*_(1mm)_	0.764 (0.681–0.834)	>2.6	78.0 (64.0–88.5)	63.3 (51.7–73.9)
*E_SD_*_(2mm)_	0.769 (0.687–0.839)	>6.2	70.0 (55.4–82.1)	68.4 (56.9–78.4)

## Data Availability

The authors confirm that the data supporting the findings of this study are available within the article.

## References

[1] Bercoff J., Tanter M., Fink M. (2004). Supersonic shear imaging: A new technique for soft tissue elasticity mapping. IEEE Trans. Ultrason. Ferroelectr. Freq. Control.

[2] Tanter M., Bercoff J., Athanasiou A., Deffieux T., Gennisson J.-L., Montaldo G., Muller M., Tardivon A., Fink M. (2008). Quantitative Assessment of Breast Lesion Viscoelasticity: Initial Clinical Results Using Supersonic Shear Imaging. Ultrasound Med. Biol..

[3] Sebag F., Vaillant-Lombard J., Berbis J., Griset V., Henry J.F., Petit P., Oliver C. (2010). Shear Wave Elastography: A New Ultrasound Imaging Mode for the Differential Diagnosis of Benign and Malignant Thyroid Nodules. J. Clin. Endocrinol. Metab..

[4] Bhatia K.S.S., Tong C.S.L., Cho C.C.M., Yuen E.H.Y., Lee Y.Y.P., Ahuja A.T. (2012). Shear wave elastography of thyroid nodules in routine clinical practice: Preliminary observations and utility for detecting malignancy. Eur. Radiol..

[5] Veyrieres J.B., Albarel F., Lombard J.V., Berbis J., Sebag F., Oliver C., Petit P. (2012). A threshold value in Shear Wave elastography to rule out malignant thyroid nodules: A reality?. Eur. J. Radiol..

[6] Park A.Y., Son E.J., Han K., Youk J.H., Kim J.-A., Park C.S. (2015). Shear wave elastography of thyroid nodules for the prediction of malignancy in a large scale study. Eur. J. Radiol..

[7] Brandenstein M., Wiesinger I., Künzel J., Hornung M., Stroszczynski C., Jung E.-M. (2022). Multiparametric Sonographic Imaging of Thyroid Lesions: Chances of B-Mode, Elastography and CEUS in Relation to Preoperative Histopathology. Cancers.

[8] Chambara N., Lo X., Chow T.C., Lai C.M., Liu S.Y., Ying M. (2022). Combined Shear Wave Elastography and EU TIRADS in Differentiating Malignant and Benign Thyroid Nodules. Cancers.

[9] Xue J.-p., Kang X.-y., Miao J.-w., Zhang Y.-x., Li H.-z., Yao F.-c., Kang C.-s. (2022). Analysis of the Influence of Thyroid Nodule Characteristics on the Results of Shear Wave Elastography. Front. Endocrinol..

[10] Moon J.H., Hwang J.-Y., Park J.S., Koh S.H., Park S.-Y. (2017). Impact of region of interest (ROI) size on the diagnostic performance of shear wave elastography in differentiating solid breast lesions. Acta Radiol..

[11] Youk J.H., Son E.J., Han K., Gweon H.M., Kim J.-A. (2017). Performance of shear-wave elastography for breast masses using different region-of-interest (ROI) settings. Acta Radiol..

[12] Bulum A., Ivanac G., Divjak E., Biondić Špoljar I., Džoić Dominković M., Bojanić K., Lucijanić M., Brkljačić B. (2021). Elastic Modulus and Elasticity Ratio of Malignant Breast Lesions with Shear Wave Ultrasound Elastography: Variations with Different Region of Interest and Lesion Size. Diagnostics.

[13] Sun Y.-M., Dong H., Du Z.-Y., Yang Z.-L., Zhao C., Chong J., Li P. (2020). The effect of regions-of-interest and elasticity modulus selection on differentiating benign and malignant cervical lymph nodes with shear wave elastography. Clinics.

[14] Lahtinen O., Pulkkinen M., Sironen R., Vanninen R., Rautiainen S. (2022). 2D-shear wave elastography in the evaluation of suspicious superficial inguinal lymph nodes: Reproducibility and region of interest selection. PLoS ONE.

[15] Filho R.H.C., Pereira F.L., Iared W. (2020). Diagnostic Accuracy Evaluation of Two-Dimensional Shear Wave Elastography in the Differentiation Between Benign and Malignant Thyroid Nodules. J. Ultrasound Med..

[16] Suh C.H., Choi Y.J., Baek J.H., Lee J.H. (2017). The diagnostic performance of shear wave elastography for malignant cervical lymph nodes: A systematic review and meta-analysis. Eur. Radiol..

[17] Yeon E.K., Sohn Y.-M., Seo M., Kim E.-J., Eun Y.-G., Park W.S., Yun S.J. (2020). Diagnostic performance of a combination of shear wave elastography and B-mode ultrasonography in differentiating benign from malignant thyroid nodules. Clin. Exp. Otorhinolaryngol..

[18] Kim H.J., Kwak M.K., Choi I.H., Jin S.Y., Park H.K., Byun D.W., Suh K., Yoo M.H. (2019). Utility of shear wave elastography to detect papillary thyroid carcinoma in thyroid nodules: Efficacy of the standard deviation elasticity. Korean J. Intern. Med..

[19] Han R.J., Du J., Li F.H., Zong H.R., Wang J.D., Shen Y.L., Zhou Q.Y. (2019). Comparisons and Combined Application of Two-Dimensional and Three-Dimensional Real-time Shear Wave Elastography in Diagnosis of Thyroid Nodules. J. Cancer.

[20] Chung S.R., Ahn H.S., Choi Y.J., Lee J.Y., Yoo R.-E., Lee Y.J., Kim J.Y., Sung J.Y., Kim J.-h., Baek J.H. (2021). Diagnostic Performance of the Modified Korean Thyroid Imaging Reporting and Data System for Thyroid Malignancy: A Multicenter Validation Study. Korean J. Radiol..

[21] Foncea C.G., Popescu A., Lupusoru R., Fofiu R., Sirli R., Danila M., Sporea I. (2020). Comparative study between pSWE and 2D-SWE techniques integrated in the same ultrasound machine, with Transient Elastography as the reference method. Med. Ultrason..

[22] Kang H.-J., Lee J.Y., Lee K.B., Joo I., Suh K.-S., Lee H.-K., Han J.K. (2019). Addition of Reliability Measurement Index to Point Shear Wave Elastography: Prospective Validation via Diagnostic Performance and Reproducibility. Ultrasound Med. Biol..

[23] Mulabecirovic A., Mjelle A.B., Gilja O.H., Vesterhus M., Havre R.F. (2018). Repeatability of shear wave elastography in liver fibrosis phantoms—Evaluation of five different systems. PLoS ONE.

[24] Kiwan C., Donggeon K., Zaegyoo H., Hyoung-Ki L. A reliability index of shear wave speed measurement for shear wave elastography. Proceedings of the 2015 IEEE International Ultrasonics Symposium (IUS).

[25] Öztürk V.S., Ertekin E. (2021). Diagnostic performance of shear wave elastography and diffusion-weighted magneticresonance imaging in cervical lymph nodes: A comparative study. Turk. J. Med. Sci..

[26] Oruk Y.E., Çildağ M.B., Karaman C.Z., Çildağ S. (2021). Effectiveness of ultrasonography and shear wave sonoelastography in Sjögren syndrome with salivary gland involvement. Ultrasonography.

[27] Duan S.B., Yu J., Li X., Han Z.Y., Zhai H.Y., Liang P. (2016). Diagnostic value of two-dimensional shear wave elastography in papillary thyroid microcarcinoma. OncoTargets Ther..

[28] Prasad S.B., Lin A.K., Guppy-Coles K.B., Stanton T., Krishnasamy R., Whalley G.A., Thomas L., Atherton J.J. (2018). Diastolic Dysfunction Assessed Using Contemporary Guidelines and Prognosis Following Myocardial Infarction. J. Am. Soc. Echocardiogr..

[29] Koo T.K., Li M.Y. (2016). A Guideline of Selecting and Reporting Intraclass Correlation Coefficients for Reliability Research. J. Chiropr. Med..

[30] Dobruch-Sobczak K., Zalewska E.B., Gumińska A., Słapa R.Z., Mlosek K., Wareluk P., Jakubowski W., Dedecjus M. (2016). Diagnostic Performance of Shear Wave Elastography Parameters Alone and in Combination with Conventional B-Mode Ultrasound Parameters for the Characterization of Thyroid Nodules: A Prospective, Dual-Center Study. Ultrasound Med. Biol..

[31] He Y.-P., Xu H.-X., Wang D., Li X.-L., Ren W.-W., Zhao C.-K., Bo X.-W., Liu B.-J., Yue W.-W. (2017). First experience of comparisons between two different shear wave speed imaging systems in differentiating malignant from benign thyroid nodules. Clin. Hemorheol. Microcirc..

[32] Tan S., Sun P.-F., Xue H., Fu S., Zhang Z.-P., Mei F., Miao L.-Y., Wang X.-H. (2021). Evaluation of thyroid micro-carcinoma using shear wave elastography: Initial experience with qualitative and quantitative analysis. Eur. J. Radiol..

[33] Yoo H.W., Kim S.G., Jang J.Y., Yoo J.J., Jeong S.W., Kim Y.S., Kim B.S. (2022). Two-dimensional shear wave elastography for assessing liver fibrosis in patients with chronic liver disease: A prospective cohort study. Korean J. Intern. Med..

[34] Foncea C., Popescu A., Lupusoru R., Cotrau R., Bende F., Moga T., Sirli R., Sporea I. (2022). PErformance of a 2D-SWE Method for the Diagnosis of Liver Fibrosis Using Transient Elastography as Reference Method. Ultrasound Med. Biol..

[35] Yoo J., Lee J.M., Joo I., Yoon J.H. (2020). Assessment of liver fibrosis using 2-dimensional shear wave elastography: A prospective study of intra- and inter-observer repeatability and comparison with point shear wave elastography. Ultrasonography.

[36] Haugen B.R., Alexander E.K., Bible K.C., Doherty G.M., Mandel S.J., Nikiforov Y.E., Pacini F., Randolph G.W., Sawka A.M., Schlumberger M. (2016). 2015 American Thyroid Association Management Guidelines for Adult Patients with Thyroid Nodules and Differentiated Thyroid Cancer: The American Thyroid Association Guidelines Task Force on Thyroid Nodules and Differentiated Thyroid Cancer. Thyroid Off. J. Am. Thyroid Assoc..

[37] Kleiman D.A., Beninato T., Soni A., Shou Y., Zarnegar R., Fahey T.J. (2013). Does Bethesda Category Predict Aggressive Features in Malignant Thyroid Nodules?. Ann. Surg. Oncol..

